# Perinatal tissue-derived exosomes ameliorate colitis in mice by regulating the Foxp3 + Treg cells and gut microbiota

**DOI:** 10.1186/s13287-023-03263-1

**Published:** 2023-03-20

**Authors:** Yaping Yan, Kaixiu Li, Jiang Jiang, Lihong Jiang, Xiang Ma, Fang Ai, Shuai Qiu, Wei Si

**Affiliations:** 1 State Key Laboratory of Primate Biomedical Research, Institute of Primate Translational Medicine, Kunming University of Science and Technology, Kunming, 650500 Yunnan China; 2 Department of Obstetrics, The First People’s Hospital of Yunnan Province, Kunming, 650032 Yunnan China; 3 Affiliated Hospital of Kunming University of Science and Technology, Kunming, 650032 Yunnan China; 4 Yunnan Key Laboratory of Innovative Application of Traditional Chinese Medicine, The First People’s Hospital of Yunnan Province, Kunming, 650032 Yunnan China

**Keywords:** IBD, Exosomes, Foxp3 + Treg cells, Inflammatory, Gut microbiota

## Abstract

**Background:**

The capacity of self-renewal and multipotent differentiation makes mesenchymal stem cells (MSC) one of the most widely investigated cell lines in preclinical studies as cell-based therapies. However, the low survival rate and poor homing efficiency of MSCs after transplantation hinder the therapeutic application. Exosomes derived from MSCs have shown promising therapeutic potential in many diseases. However, the heterogeneity of MSCs may lead to differences in the function of secreting exosomes. In this study, the therapeutic effects of hUC-Exos and hFP-Exos on the DSS-induced colitis mouse model were investigated.

**Methods:**

The colitis mouse models were randomly divided into four groups: (1) DSS administered for 7 days and euthanasia (DSS7D), (2) DSS administered for 7 days and kept for another 7 days without any treatment (DSS14D), (3) DSS administered for 7 days and followed with hUC-EVs infusion for 7 days (hUC-EVs) and (4) DSS administered for 7 days and followed with hFP-EVs infusion for 7 days (hFP-EVs). We analyzed colon length, histopathology, Treg cells, cytokines and gut microbiota composition in each group.

**Results:**

A large amount of IL-6, IL-17 and IFN-γ were produced along with the decrease in the number of CD4 + Foxp3 + and CD8 + Foxp3 + cells in DSS7D group, which indicated that Th17 cells were activated and Treg cells were suppressed. We found that the number of CD4 + Foxp3 + and CD8 + Foxp3 + cells increased in order to suppress inflammation, but the length of colon did not recover and the symotoms were worsened of the colonic tissue in DSS14D group. The subsequent infusion of either hUC-Exos or hFP-Exos mediated the transformation of Treg and Th17 cells in colitis mice to maintain immune balance. The infusion of hUC-Exos and hFP-Exos also both reduced the abundance of pro-inflammatory intestinal bacterial such as Verrucomicrobia and *Akkermansia muciniphila* to improve colitis.

**Conclusions:**

We found that Foxp3 + Treg cells can inhibit the inflammatory response, and the over-activated Treg cells can still further damage the intestinal mucosa. hUC-Exos and hFP-Exos can control inflammation by regulating the balance between Th17 cells and Treg cells. Decreased inflammatory response improved the structure of colon wall in mice and reduced the abundance of pro-inflammatory bacteria in the intestine. The improvement of intestinal wall structure provides conditions for the reproduction of beneficial bacteria, which further contributes to the reduction of colitis.

**Supplementary Information:**

The online version contains supplementary material available at 10.1186/s13287-023-03263-1.

## Background

Inflammatory bowel disease (IBD) including Crohn’s disease (CD) and ulcerative colitis (UC) is a chronic and lifelong pathological condition accompanied by severe tissue destructive lesions. The exacerbation of IBD occurs when immune system over-reacts to the bacterial community presenting in intestines and triggers a series of uncontrolled inflammatory events that can damage the intestinal wall [1]. Various factors including genetics, environment, diet and gut microbiota have been associated with the development of IBD pathology [2]. The incidence of IBD is increasing globally and has become a worldwide health issue. Currently, there is no cure for IBD and the use of anti-inflammatory drugs to slow down the disease progression is the main therapeutic strategy. However, the anti-inflammatory treatment inevitably brings side effects such as weakened immunity and drug resistance. Therefore, new therapeutic strategy is needed for the treatment of IBD [3].

During the past two decades, studies on the pathogenesis of IBD mainly focused on the unusual activation of T cells including Th1 and Th2 subsets [4]. Subsequent study demonstrated that Th17 cells and their related cytokines are also involved in the pathogenesis of IBD as mediators [5]. Th17 cells are a subset of effectors T cells that are characterized by the production of large amounts of IL-17A and possess of the pro-inflammatory capability. Th17 cells are induced by the combination of interleukin-6 (IL-6) and transforming growth factor β (TGF-β), and the expansion is promoted by interleukin- 23 (IL-23) [6]. IL-17A has been widely applied as a marker of intestinal inflammation, since higher level of IL-17A was detected in the mucosa of either CD or UC patients compared to healthy people [7, 8]. In addition to Th17 cells, the CD4 + Treg cells is another distinct regulatory subsets of T cells. Regulatory cells play a key role in maintaining immune homeostasis and self-tolerance. The CD4 + Treg cells characterized by the expression of transcription factor Foxp3 is regulated by TGF-β [9]. The balance between Th17 cells and Treg cells is critical for immune homeostasis [10]. However, the role of Treg cells in IBD is still unclear. In addition, IBD is believed to be caused by abnormal immune responses to symbiotic bacteria, which disrupt the host–microbe balance [11, 12]. Previous studies have also shown that the diversity of gut microbiota is reduced in IBD patients, and *Ruminococcus gnavus* and adherent invasive *Escherichia coli* have been linked to IBD and gut inflammation [13, 14]. Colonizing germ-free mice with fecal microbiota from IBD patients can increase the number of Th17 cells, and *Akkermansia muciniphila* can induce T cells to differentiate into Th17 cells in the context of inflammation [15].

Mesenchymal stem cells have been widely used in practice of preclinical studies and clinical trials in the treatment of various diseases [16–18]. Clinical trials in CD patients showed that MSCs activated the production of Th17/Th1 cells, while they inhibited the production of Foxp3 + Treg cells. As a result, the balance between Th17/Th1 cells and Treg cells was reestablished [19, 20]. However, intravenously infused MSCs in rodents are mostly trapped in the capillaries of lung and are subsequently cleared [21, 22]. The low survival rate, poor homing efficiency and differentiating ability of MSCs after transplantation hinder the therapeutic application of MSCs [23]. MSCs secrete a wide range of paracrine factors responsible for up to 80% of their therapeutic effect [24]. Exosomes are one of the paracrine factors secreted by MSCs, which has been considered as the central mediator of intercellular communication via transferring proteins, mRNAs and miRNAs to neighboring cells [25, 26]. MSC-derived exosomes mimic the ability of MSCs to regulate the activity of immune cells including T, B, dendritic and macrophages cells [27]. The application of exosomes instead of MSCs can avoid the risk of thrombosis and tumorigenicity. However, MSCs derived from different tissues showed different biological characteristics that reflected through their different modes of action, such as proliferative, immunological, transdifferential and paracrine [28, 29]. For example, the micRNA expression profiles between adipose- and bone marrow MSC-isolated exosomes existed striking differences in conserved tRNA [30]. The heterogeneity of MSCs derived from various tissues may lead to differences in the function of secreting exosomes. Therefore, in order to achieve the preferable therapeutic effects by the application of MSCs or exosomes to treat diseases such as colitis, the function of cells and exosomes should be evaluated and the most suitable type of cell source should be determined.

The mouse models with colitis are induced by dextran sodium sulfate (DSS) that has been widely used in the pathogenetic study and therapeutic strategy development [31]. MSC-derived exosomes have showed great promising to cure colitis either in preclinical or clinical practices. The prenatal tissues mainly include umbilical cord (UC) and fetal placenta (FP) are the most primary source for derivation of MSCs due to the abundant source and noninvasive harvest process. However, whether the exosomes derived from UC-MSCs and FP-MSCs have therapeutic effects on IBD treatment is unclear. In this study, we derived exosomes from UC-MSCs and FP-MSCs isolated from the same donor’s prenatal tissues and compared the therapeutic effects on DSS-induced mouse models with colitis evaluated by pathology. We also explored the effect of exosomes derived from UC-MSCs and FP-MSCs on the regulation of T cells, inflammation and the homeostasis reestablishment of gut microbiota. The present preclinical study is beneficial to understand the interaction between exosomes and intestinal microbiota by the regulation of internal inflammation, and provide a new stragety for the treatment of IBD.

## Methods

### Animals

Four-week-old BALB/c mice (Charles River, Beijing, China) with body weight between 20 and 30 g were administrated with DSS to induce colitis model. The mice were housed in a room with a 12 h light:12 h dark cycle and provided with sterile food and water ad libitum. The temperature was controlled at 22 °C. The procedures for mice model generation and exosomes injection were approved by the Institutional Animal Care and Use Committee of Kunming University of Science and Technology and were performed in accordance with the Guide for the Care and Use of Laboratory Animals (PZWH-K2022-0021). All of the chemicals used in this study were obtained from Sigma Chemical Co. (St. Louis, MO, USA), unless otherwise indicated.

### Cell isolation and expansion

Usage of human sample (*n* = 3, from healthy donors) and the protocol used in this study are approved by Ethics Committee of Kunming University of Science and Technology (KMUST-MEC-195). Umbilical cord and placenta were harvested from fetuses with cesarean from the First People's Hospital of Yunnan Province. All the donors (*n* = 3) provided informed consents prior to tissue donation. UC and FP were cleaned with physiological saline. FP was cut into small pieces about 1 cm in diameter. UC was cut into small pieces about 1 cm in diameter after the blood vessel was removed. The tissue pieces were cultured in a Petri dish with low-glucose DMEM supplemented with 12% fetal bovine serum, 100 U/mL penicillin and 100 U/mL streptomycin. The medium was changed every 3 days. When the cells reach about 80% confluence, they were passaged and cultured.

### Flow cytometric analysis for surface marker profiles of MSCs

Expression of cell surface markers on either UC-MSCs or FP-MSCs was examined by flow cytometry using a commercial MSC Analysis Kit (BD Biosciences, San Jose, CA, USA) according to the manufacturer’s instructions as described previously [32]. Briefly, 5 × 10^5^ MSCs were collected, washed and centrifuged in 300 μL of PBS. MSCs were then resuspended in 100 μL of PBS and incubated with antibodies on ice to detect cell surface markers including CD44, CD90, CD73 and negative cocktail (CD34, CD11b, CD19, CD45, HLA-DR). Unbound antibodies were washed away by centrifugation, and the cells were resuspended in 300 μL of PBS and examined via flow cytometric analysis.

### Evaluation of the differentiation potential of MSCs

The differentiation capacity of MSCs was conducted using the Tri-lineage Differentiation Kit (Gibco BRL, Grand Island, NY, USA) as described previously [32]. For osteogenic differentiation, MSCs were seeded into 24-well plates and cultured with an osteogenic differentiation medium (Gibco) for 21 days. The osteogenic differentiation was confirmed by the appearance of Alizarin Red stain. For adipogenic differentiation, MSCs were cultured in an adipogenic differentiation medium (Gibco) for 7 days. The adipogenic differentiation was confirmed by the cellular accumulation of neutral lipid vacuoles, which were stained red with Oil Red O. For chondrogenic differentiation, MSCs were collected in 15-mL centrifuge tubes and cultured in a chondrogenic differentiation medium (Gibco). After 21 days of differentiation induction, the pellets were sectioned, and then, sulfated proteoglycans were visualized by staining with 1% toluidine blue (Merck, Darmstadt, Germany) for 10 min. The chondrogenic differentiation was confirmed by the appearance of Alcian Blue stain.

### Extraction and identification of exosomes derived from MSCs

MSC-conditioned medium (Exo-depleted medium, ViVaCell, BI, China) was collected after the cells reach 80% confluence followed by a centrifugation at 300 g for 10 min. Briefly, the dead cells in the supernatant were removed by centrifuge at 2500 g for 25 min. The cell debris in the supernatant were removed by centrifuge at 10,000 g for 30 min. Then, the supernatant was ultracentrifuged (himac CP100WX, HITACHI, Japan) at 100,000 g for 140 min at 4 °C to pellet the exosomes. The pellet was then washed once by mixing with an appropriate amount of PBS and centrifuge at 100,000 g at 4 °C for 70 min. The concentration of each exosomes was determined by a bicinchoninic acid (BCA) kit (Abcam, Cambridge, MA, USA). The morphologic characteristics of MSC-Exos were observed by Nanoparticle Tracking Video Microscope PMX-120 (NTA). The phenotypic profile of MSC-Exos was determined by western blot with CD63 (1:500, Millipore), TSG101 (1:1000, Millipore) [33, 34]. Briefly, total protein was separated by a sodium dodecyl sulfate–polyacrylamide gel electrophoresis (SDS-PAGE) gel under denaturing conditions and then transferred to polyvinylidene difluoride (PVDF) membranes (Millipore Sigma, MA, USA). Membranes were blocked with 5% skimmed milk for 1 h, followed by the incubation of specific primary antibodies (Cell Signaling Technology, Danvers, MA, USA) overnight. After being washed with Tris-buffered saline and tween 20 (TBST, Yeasen, Shanghai, China) for 3 times, membranes were incubated with the secondary antibody (Cell Signaling Technology, Danvers, MA, USA) at room temperature for 1 h. Immunoreactive bands were exposed by enhanced chemiluminescence method, and the relative protein expression levels were reflected by target protein/reference β-actin.

### Quantification and preservation of exosomes

The concentration of each exosomes was determined by a BCA kit. Dispense the exosomes into sterile EP tubes, each EP tube contains 50 µg of exosome protein. Finally, put the EP tube containing the exosomal protein in a − 80 °C refrigerator and keep it for no more than 2 months.

### DSS-induced colitis mouse models

Mice were anesthetized using isoflurane (RWD lifescience, China), which placing the mice in a closed container permeated with a mixture of isoflurane and oxygen vapor. DSS (36,000–50,000 molecular weight; MP Biomedicals) was diluted to 3% with deionized water before use, and the mice were intragastrically administered solution of daily up to 7 days. The mice with free access for sterile food and water were served as control and were euthanized on the 7th day to collect samples. The IBD mouse models were randomly divided into four groups: (1) DSS administered for 7 days and euthanasia (DSS7D group), (2) DSS administered for 7 days and kept for another 7 days without any treatment (DSS14D group), (3) DSS administered for 7 days and followed with hUC-Exos infusion for 7 days (hUC-Exos group) and (4) DSS administered for 7 days and followed with hFP-Exos infusion for 7 days (hFP-Exos group). Dissolve 50 µg exosomes protein in 200 µl of normal saline and injected into mice via tail vein per day. At the end of treatments, the mice in each group were euthanized for subsequent analysis. Briefly, mice were killed using CO_2_ at 20% chamber replacement rate. Intact colons from the epityphlon to the anus were harvested, and colon lengths were measured. Photographs of the colon were acquired at a vertical angle immediately after the samples were harvested.

### Exosome labeling and tracking in vivo

Exosomes (about 1 µg/µL at protein level) were incubated with 1 mM DiR (Invitrogen) at the volume ratio of 500:1 for 30 min. About 100 μg of DiR-labeled exosomes were tail vein-injected into mice. Exosome localization in the whole body and individual organs was detected by IVIS ® Lumina II in *vivo* imaging system (PerkinElmer, Thermo Fisher, USA) on day 7 after injection.

### Flow cytometry of Foxp3 + cells

Immune cells are tested on the 7th and 14th days. Peripheral blood mononuclear cell (PBMC) isolated from peripheral blood was stained with fluorescence-labeled antibodies (CD4-FITC, CD8-APC and Foxp3-PE, BioLegend, USA) followed by the wash of cold PBS. Flow cytometric analysis was performed on a BD FACSCalibur flow cytometer (FACSAria II, BD, USA), and the data were analyzed with Flowjo VX software.

### Cytokine detection by cytometric bead array

Cytokine are tested on the 7th and 14th days in each group. Prior to blood collection, mice were fasted for eight hours. The collected serum samples were stored at − 80 °C. IL-6, IFN-γ, TNF-α, IL-17A, IL-10 and TGF-β were tested using a BD Cytometric Bead Array (CBA) Mouse Th1/Th2/Th17 Cytokine Kit according to manufacturer’s instructions. The mouse Th1/Th2/Th17 Cytokine data were analyzed by using FCAP Array software (BD Biosciences).

### RNA isolation and qRT-PCR

The total RNA of the colon was extracted by using TRIzol (Invitrogen, USA) according to manufecture’s instructions. Approximate 1 µg of RNA was reverse transcribed into cDNA using miRcute Plus miRNA qPCR Detection Kit (TIANGEN, Beijing, China). qPCR was performed with SYBR Green PCR master mix (Roche, Switzerland). Relative expression of mRNA was normalized to β-actin and calculated by 2 − ΔΔCt. The PCR primer sequences are listed in Additional file 1: Table S1.

### Hematoxylin and eosin (H&E) staining

The dissected colon tissues were fixed in 4% formalin (Thermo Fisher Scientific, MA, USA) for 24 h, embedded into paraffin (Thermo Fisher Scientific) and sliced into 5-μm sections. The slides were stained with hematoxylin and eosin (Sigma-Aldrich) and examined with a light microscope. The histological scores were evaluated by observers who were blinded as to which treatment group the mice belonged to. The histological damage score is the sum of evaluation based on crypt architecture, mucosal damage and muscle thickening of the tissue [35].

### 16S rRNA sequencing of fecal microbiota

Contents of colon were collected directly from the mice in each group were euthanized. Fecal samples were then stored at − 80 °C for further analysis. The genomic DNA from the total 50 samples was extracted using a QIAamp Fast DNA Stool Mini kit (Qiagen, Germany) according to the manufacturer’s instructions. The quality and quantity of genomic DNA were then determined by 1% agarose gel electrophoresis and using a NanoDrop 8000 instrument (Thermo, USA). The V3–V4 region of the bacterial 16 s rRNA gene was amplified by polymerase chain reaction (PCR) using the primers 338F (5′-GTACTCCTACGGGAGGCAGCA-3′) and 806R (5′-GTGGACTACHVGGGTWTCTAAT-3′), followed by 8-bp unique barcode sequencing performed by Illumina Miseq PE300 sequencing. The PCR protocol involved an initial denaturation at 94 °C for 4 min; 25 cycles of denaturation at 94 °C for 45 s, annealing at 55 °C for 50 s and extension at 72 °C for 45 s; and then a final extension at 72 °C for 10 min.

### Statistical analysis

All data are expressed as the mean ± SEM. Statistical significances were analyzed by SPSS (version 16.0) using the one-way ANOVA. Differences with *p* < 0.05 were considered statistically significant.

## Results

### Extraction and characterization of Exos from UC-MSCs and FP-MSCs

**Fig. 1:**
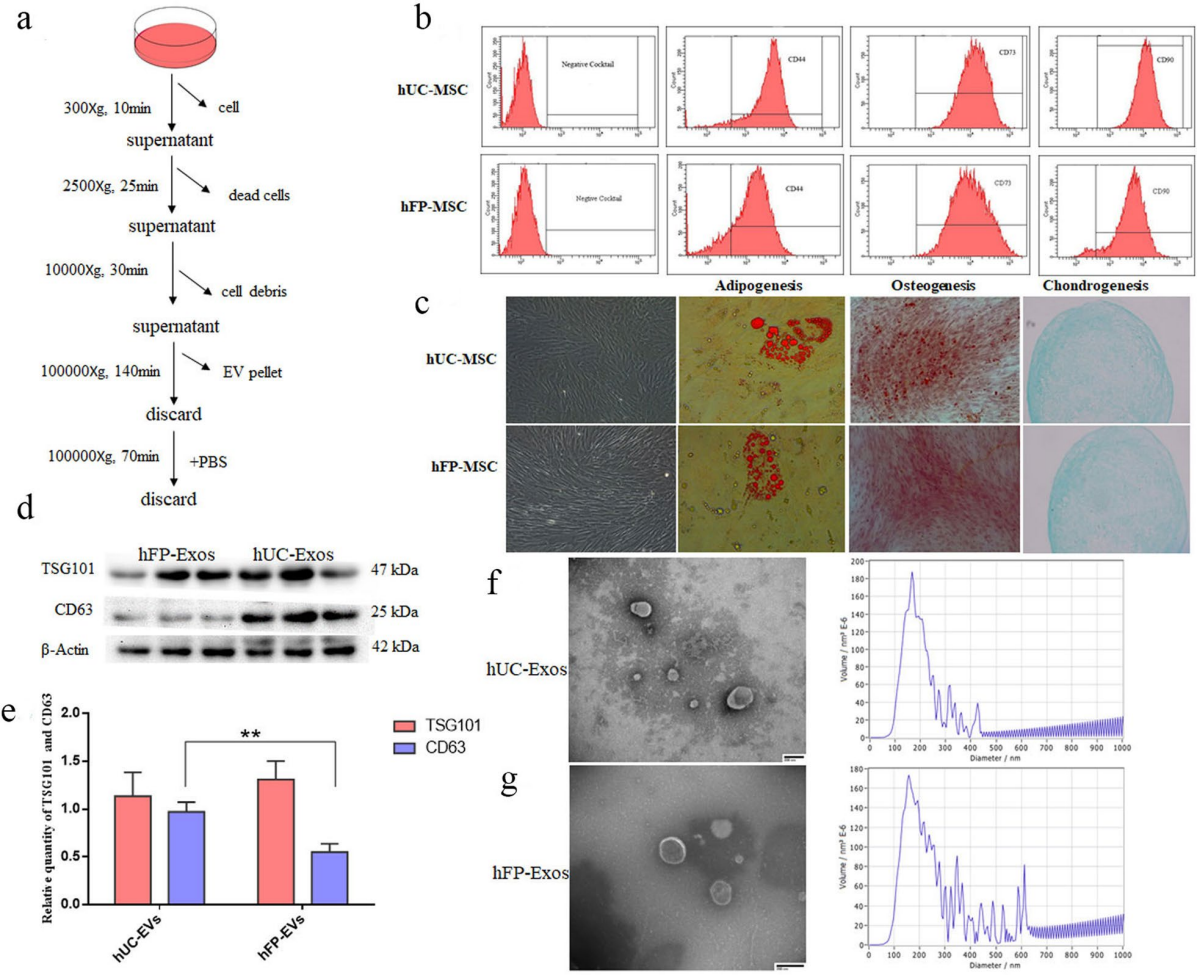
Extraction and identification of hUC-Exos and hFP-Exos. **a** Extraction of exosomes by ultracentrifugation. **b**, **c** Characterization of surface markers and tri-lineage differentiating capability of hUC-MSCs and hFP-MSCs. **d** Exosome marker proteins (TSG101and CD63) in hUC-Exos and hFP-Exos analyzed by western blotting. Full-length blots are presented in Additional file 2: Fig. S1. **e** Integrated optical densities of TSG101 and CD63 between the hUC-Exos and hFP-Exos. **f**, **g** Transmission electron microscopy images of hUC-Exos and hFP-Exos. Scale bars = 200 µm

The procedure for extraction of exosomes from hUC-MSCs and hFP-MSCs by ultracentrifugation is described in Fig. 1a. The surface marker profiles of UC- and FP-MSCs were analyzed at passage 4 by flow cytometry. Both hUC-MSCs and hFP-MSCs express positive surface markers at high ratios, which include CD44, CD73 and CD90 but barely expressed the cocktailed negative surface markers (Fig. 1b). The cells also presented the capability of adipogenic, osteogenic and chodrogenic differentiation (Fig. 1c). Both hUC-MSCs and hFP-MSCs were expanded in Exo-depleted medium. Both hUC-Exos and hFP-Exos were positive for TSG101 and CD63 (Fig. 1d, e), and exhibited similar morphology and size (diameter > 100 nm) (Fig. 1f, g).

### hUC-Exos and hFP-Exos alleviated DSS-induced colitis of mice

**Fig. 2:**
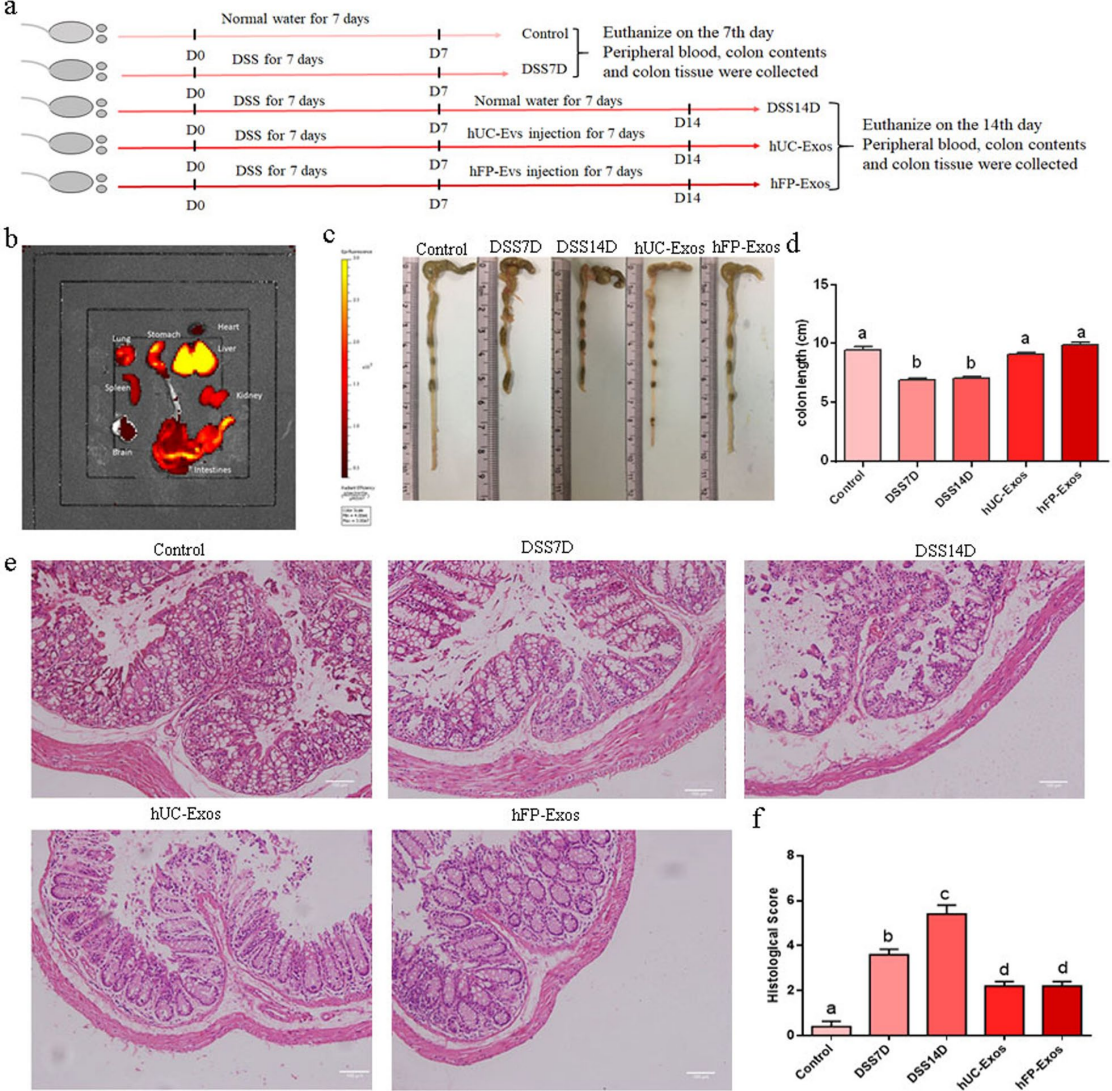
Exosomes alleviated DSS-induced mouse colitis. **a** Experimental procedure. **b** Imaging of DiR-labeled exosomes from different tissues. **c**, **d** Effects of DSS administration and hUC-Exos or hFP-Exos treatment on the colon length of colitis mouse models. **e**, **f** HE staining and histopathological score of colon tissues. hUC-Exos and hFP-Exos administration significantly reduced histopathological score of the DSS mice including inflammatory cell infiltration and crypt architecture reduce. The statistical results are collected from 10 mice in each group. Different letters on the columns indicate significant differences

The experimental procedure is illustrated in Fig. 2a. After intragastrically administered 3% DSS for 7 days, mice displayed symptoms of acute colitis. Notably, the exosomes showed high ability to circulate into liver, stomach, colon and rectum (Fig. 2b). We evaluated the colon length of the mice from the 5 groups. The colon length of DSS7D group was significantly shorter than control group, and the colon length of DSS14D group did not show any recovery one week after the cease of DSS administration. In contrast, the length of the colon was significantly restored after the administration of hUC-Exos and hFP-Exos (Fig. 2c, d). Colons in DSS7D groups exhibited mucosal damage and crypt architecture reduction of the tissue, and the symptoms were significantly worsened one week after the cease of DSS administration in DSS14D group. However, these symptoms were significantly alleviated 7 days after the treatment of either hUC-Exos or hFP-Exos (Fig. 2e, f).

### hUC-Exos and hFP-Exos regulate the secretion of cytokines in colitis mice

**Fig. 3:**
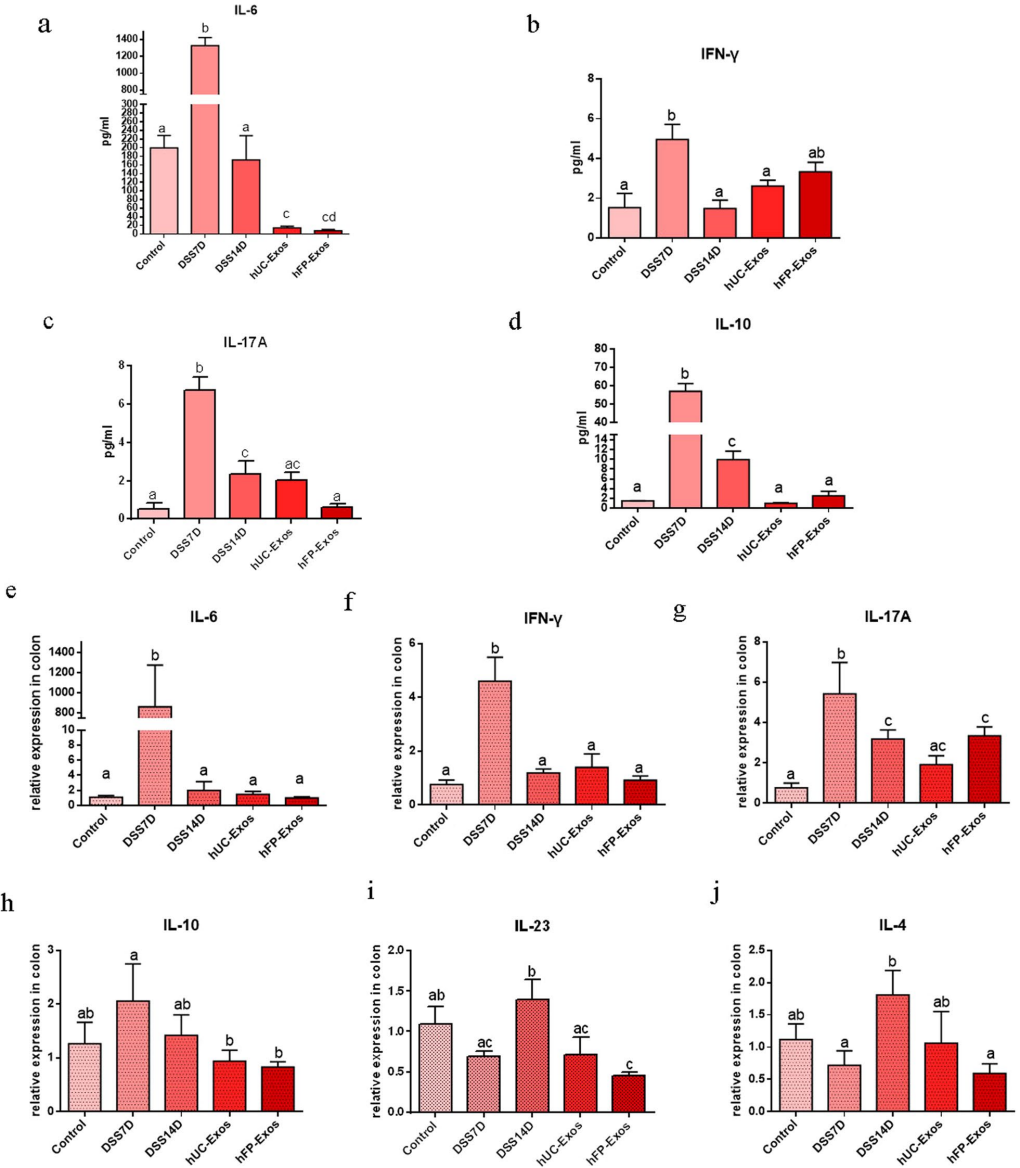
Concentration of inflammatory cytokines in peripheral blood and the correspondent gene expression in colon tissues. **a**–**d** Concentration of IL-6, IFN-γ, IL-17A and IL-10 in peripheral blood from the 5 groups. The statistical results are collected from 3 to 5 mice each group. **e**–**j** Level of related gene expression in colon tissues including IL-6, IFN-γ, IL-17A, IL-10, IL-23, IL-4. The statistical results are collected from 3 to 5 mice each group. Different letters on the columns indicate significant differences

We detected the concentration of cytokines in the peripheral blood of mice. We found that IL-6, IFN-γ, IL-17A and IL-10 greatly increased in the DSS7D group. The concentration of IL-6, IFN-γ, IL-17A and IL-10 significantly decreased in the DSS14D group compared to DSS7D group. However, the concentration of IL-6, IFN-γ, IL-17A and IL-10 were significantly returned to normal levels after the administration of hUC-Exos and hFP-Exos (Fig. 3a–d).

**Fig. 4:**
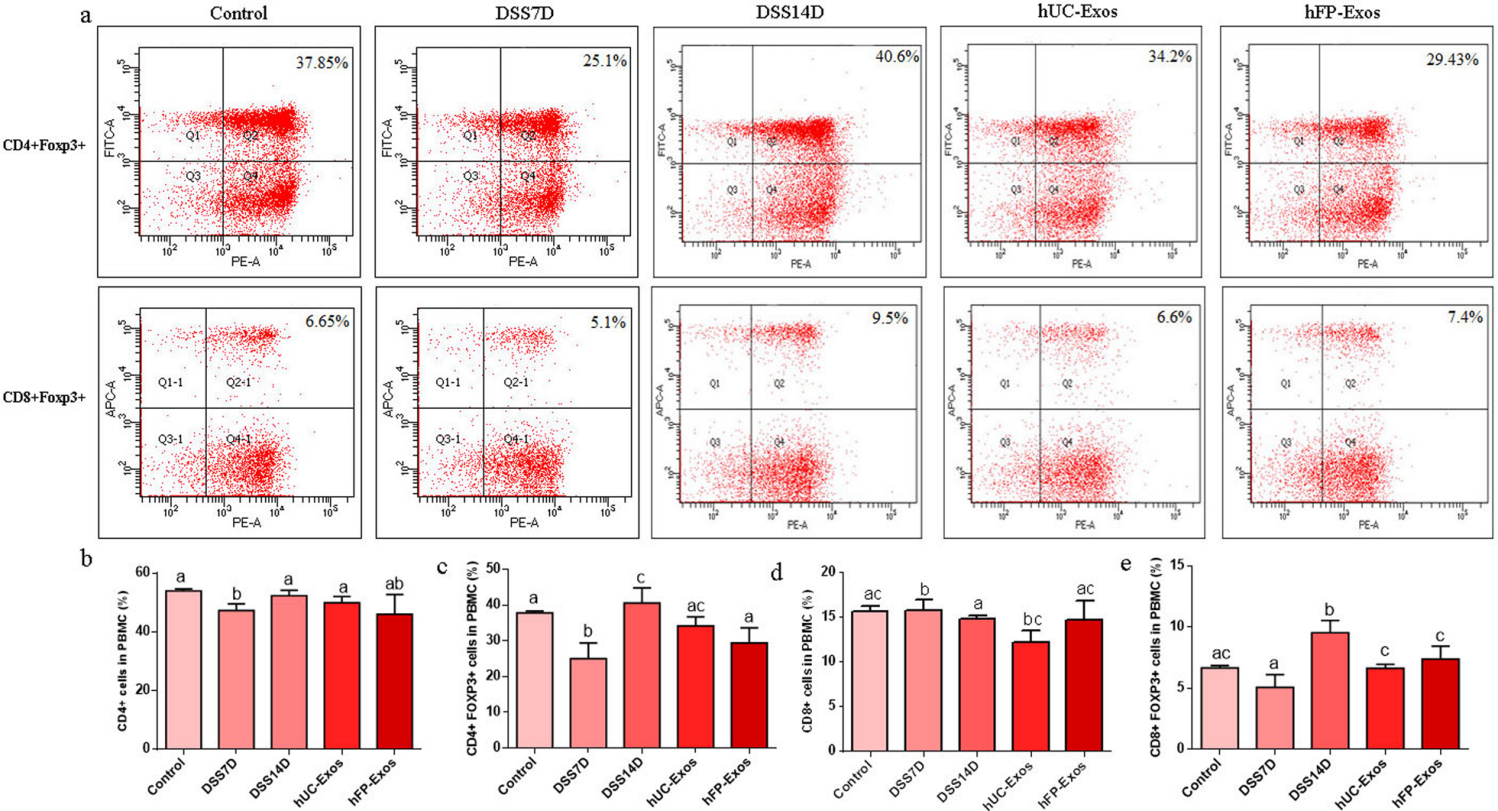
Analysis of Foxp3 + Treg cells in the five groups by flow cytometry. **a** Flow cytometry of CD4 + Foxp3 + Treg cells and CD8 + Foxp3 + Treg cells. **b** Numbers of CD4 + T cells in the five groups. **c** Numbers of CD4 + Foxp3 + Treg cells. **d** Numbers of CD8 + T cells. **e** The numbers of CD8 + Foxp3 + Treg cells. The statistical results are collected from 2 to 5 mice in each group. Different letters on the columns indicate significant differences

We also measure the mRNA expression of cytokines including IL-6, IFN-γ, IL-17A, IL-10, IL-23 and IL-4. We found that the expression of IL-6, IFN-γ, IL-17A and IL-10 in the colon significantly increased in DSS7D group, and then decreased after the infusion of hUC-Exos and hFP-Exos (Fig. 3e–h). We also found that the expression of IL-23 and IL-4 in the colon is greatly increased in DSS14D group (Fig. 4i, j). After exosomal infusion, the mice from both hUC-Exos and hFP-Exos groups showed reduced secretion of cytokines. Therefore, the infusion of either hUC-Exos or hFP-Exos remodels the immune balance of colitis.

### hUC-Exos and hFP-Exos mediate the transformation of Treg and Th17 cells in colitis mice

We investigated the number of Foxp3 + cells in the five groups and found that the number of CD4 + Foxp3 + cells and CD8 + Foxp3 + cells increased significantly in the DSS14D group, which indicated that a large number of Treg cells were produced at this stage. We also found that a large amount of IL-17A was produced in the DSS7D group. Therefore, a large number of Treg cells were produced in order to suppress inflammation after the cease of DSS administration at 7 days. However, pathological examination found that the symptom in the DSS14D group was aggravated. Therefore, over-activated Treg cells failed to alleviate the development of colitis in mice by inhibiting inflammation. The number of CD4 + Foxp3 + cells and CD8 + Foxp3 + cells decreased after the infusion of UC-Exos or FP-Exos via tail vein compared to DSS14D group (Fig. 4a–e). hUC-Exos and hFP-Exos can mediate the transformation of T cells into Treg and Th17 cells in colitis mice.

### hUC-Exos and hFP-Exos treatment alters the composition of gut microbiota

**Fig. 5:**
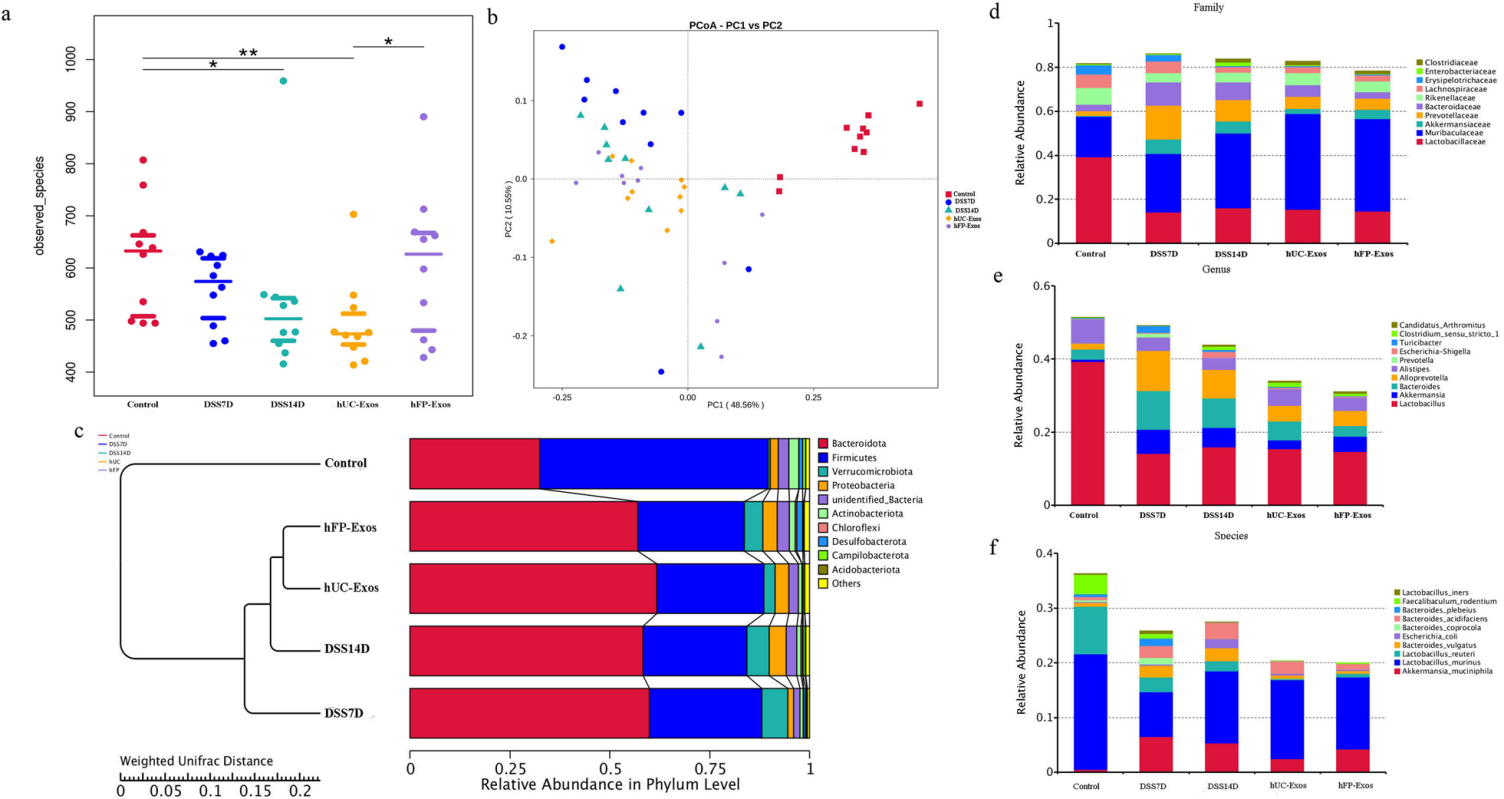
Diversity and composition analysis of gut microbiota from the five groups. **a** Alpha diversity (Shannon index) analysis. **b** PCoA of the composition of gut microbiota. **c** Evolutionary analysis of gut microbiota. **d**–**f** Composition of intestinal microbiota in family, genus and species levels. The statistical results are from 10 mice in each group

The alpha diversity decreased in DSS7D and DSS14D groups compared to the control group. The alpha diversity increased in hFP-Exos group compared to the DSS group and hUC-Exos group (Fig. 5a). The microbial composition of the other four groups is quite different compared to the control group. The composition of microbita is relatively similar between DSS7D group and DSS14D group, and the composition of microbiota is similar in hUC-Exos and hFP-Exos (Fig. 5b, c). The relative abundance of Bacteroidota increased in the other four groups except control group. Verrucomicrobiota significantly increased in DSS7D group, but subsequently decreased after the infusion of hUC-Exos and hFP-Exos. The relative abundance of Desulfobacterota enhanced in the hFP-Exos group (Fig. 5d). Lactobacillaceae and *Lactobacillus* was significantly reduced in the four experimental group compared to control group. *Akkermansia* and *Alloprevotella* decreased after the treatment of hUC-Exos and hFP-Exos compared to DSS group (Fig. 5e). The abundance of *Escherichia coli* and *Bacteroides vulgates* significantly increased in the DSS7D group and DSS14D group and reduced after the infusion either of hUC-Exos or hFP-Exos (Fig. 5f).

## Discussion

The therapeutic effect of MSC transplantation is poor, and the survival time of inoculated MSCs in the inflamed intestine is very short [36, 37], which limit the application of MSC in IBD treatment. However, exosomes isolated from MSCs show excellent therapeutic effects in animal disease models [38]. Previous studies have shown that exosomes injected into colitis mice were found in the liver, spleen and damaged colon at 12 h, 24 h and 48 h, which indicates that exosomes can reach the damaged colon and improve colitis [39–42]. However, it is unknown whether the exosomes can exist in colitis mice for a long time. In our study, we chose to analyze the therapeutic effects of exosomes after 7 days of continuous injection. Our results found that exosomes were mainly distributed in the liver, stomach and colorectum of mice at the seventh day after exosome injection, which was similar to previous reports.

After the infusion of hUC-Exos or hFP-Exos, the length of the colon of DSS mice significantly recovered, and both epithelial cells and crypts were significantly improved by pathological evaluation. In order to exclude the possible self-recovery of the colon after 7 days of DSS administration, we also set up the DSS14D group. We found that the colon length of mice did not recover in the DSS14D group, and more importantly, the number and morphology of crypts in DSS14D mice decreased sharply compared to DSS7D group.

Previous studies have proved that IL-17A secretion significantly increased in IBD patients [7, 8]. It is also known that Th17 cells express the pro-inflammatory cytokine IL-17 and IFN-γ, which plays an important role in the pathogenesis of inflammatory diseases. In our results, we found that the secretion of IL-17A, IL-6 and IFN-γ significantly increased in the DSS7D group. The results are in agreement with those observed in IBD patients [43, 44]. Initial CD4 + T cells differentiate into Th17 cells in response to IL-6 and become more pathogenic in the presence of IL-23 [10, 45, 46]. IL-23 can induce and exacerbate intestinal inflammation [47]. IL-6 acts as a potent pro-inflammatory cytokine in T cells through promotion of Th17 differentiation and inhibiting Treg cell differentiation. These evidences indicate that T cells mainly differentiate into Th17 cells in the DSS7D group. Seven days after DSS treatment in DSS14D group, the mice showed a significant increase in the number of CD4 + Foxp3 + and CD8 + Foxp3 + cells, and a reduction in the secretion of IL-6 and IL-17A compared to mice in DSS7D group. Previous study found that IL-4 may be a cytokine that activates the high expression of inflammatory proteins and participates in the immune regulation of Th2 cells in patients with ulcerative colitis [48]. The expression of IL-4 was significantly increased in the DSS14D group. IL-4 can inhibit the differentiation of T cells into Th17 cells [49]. These changes indicated that the differentiation of T cells into Th17 cells was suppressed and the production of Treg cells were significantly increased. However, we did not observe the improvement of colon length and pathological symptom in the DSS14D group. Similar phenomenon was observed in the peripheral blood of patients with colorectal cancer that the number of Treg cells increased significantly [50], which may also explain the deterioration of the pathology of the mice in the DSS14D group. Therefore, although a large increase in Foxp3 + Treg cells can inhibit the inflammatory response, the over-activated Treg cells can still further damage the intestinal mucosa [51]. By infused with hUC-Exos or hFP-Exos daily for 7 days after DSS induction, mice in both hUC-Exos and hFP-Exos did not show increased secretion of IL-17A and IL-6 compared to the DSS14D groups, though the number of CD4 + Foxp3 + Treg cells was found decreased. Therefore, the results suggested that hUC-Exos and hFP-Exos mediated the balance of Th17 and Treg cells in IBD mice. We also found that the infusion of hUC-Exos and hFP-Exos regulated the level of IL-10 on mice with colitis. Since IL-10 is regarded as an anti-inflammatory cytokine and can control the Th17 prevalence [52–54], the maintenance of immune homeostasis of Th17 cells and Treg cells may be a key target for the treatment of IBD.

The diversity and instability of intestinal bacteria lose balance in IBD patients [55, 56]. However, whether the immuoregulation of MSCs or MSC-derived Exos can impact the reestablishment of healthy intestinal microbiota homeostasis is still unknown. Our results showed that alpha diversity of gut microbiota was reduces after treatment with hUC-Exos and hFP-Exos. But the alpha diversity of gut microbiota was higher in hFP-Exos group compared to hUC-Exos group. Shannon index is a comprehensive index to the abundance and uniformity of the bacterial community. The higher of Shannon index, the higher of diversity and uniformity in the microbiota community. Therefore, the greater individual difference of mice may result in higher Shannon index in hFP-Exos group. We found that pro-inflammatory bacteria did not decrease in the DSS14D group compared to the DSS7D group, but significantly decreased after hUC-Exos and hFP-Exos treatments. Verrucomicrobia is suggested to be directly related to colon inflammation [57, 58]. In our study, the treatment of either hUC-Exos or hFP-Exos decreased the relative abundance of Verrucomicrobia. The abundance of Akkermansiaceae and Akkermansia elevated in IBD patients, which can predict the effect of IBD treatment with an accuracy rate of 76.5% [59], and *Akkermansia muciniphila* can induce the activation of Th17 cells in an inflammatory environment [15]. In our results, Akkermansia and *A. muciniphila* significantly increased in DSS7D group. In contrast, the relative abundance of Akkermansiaceae, Akkermansia and *A. muciniphila* reduced after the treatment of hUC-Exos or hFP-Exos, and the scope of reduction was especially greater in the hUC-Exos group. Furthermore, the abundance of another two symbiotic bacteria (*Escherichia coli* and *Bacteroides vulgatus*) linked to IBD and inflammation [60] were observed significantly increased in the DSS7D group and DSS14D group. In contrast, the abundance of *Escherichia coli* and *Bacteroides vulgatus* was decreased after the treatment with hUC-Exos or hFP-Exos. Therefore, our results demonstrated for the first time that the infusion of hUC-Exos or hFP-Exos can rebalance IBD mouse intestinal microbiota by regulating inflammation, and in turn, the reduction of pro-inflammatory bacteria helps the healing of symptom of mice with colitis. Besides that, we speculate that exosomes mediated the balance of the number of Treg and Th17 cells, and the immune balance further promoted changes in the composition of gut microbiota. Finally, the changes in the gut microbiota further reduced the intestinal inflammation.

Severe inflammatory reaction reduced the number of colonic crypts and a large number of inflammatory cells infiltrated in mice with colitis, which led to increase the abundance of pro-inflammatory bacteria. The destruction of intestinal wall structure promotes harmful substances to enter the peripheral circulation, which further aggravates inflammation in mice. By analyzing Treg cells in the peripheral blood of mice, we found that CD4 + Foxp3 + Treg and CD8 + Foxp3 + Treg were abundant in DSS14D mice. Therefore, we speculate that over-activated Treg cells will also aggravate colitis in mice. Exosomes can control inflammation by regulating the balance between Th17 cells and Treg cells. Decreased inflammatory response improved the structure of colon wall in mice and reduced the abundance of pro-inflammatory bacteria in the intestine. The improvement of intestinal wall structure provides conditions for the reproduction of beneficial bacteria, which further contributes to the reduction of colitis.

## Conclusion

In summary, we investigated the therapeutic effects of hUC-Exos or hFP-Exos on DSS-induced mice with colitis. Our results indicated that (1) the colon of mice with colitis did not recover but showed severe pathological symptom after the cease of DSS administration up to 7 days; (2) MSC-Exos can home to injured mouse intestines through tail vein injection; (3) over-activated Treg cells aggravated the pathological symptom of mice with colitis, and hUC-Exos and hFP-Exos infusion regulated the balance of Th17 cells and Treg cells; (4) the infusion of hUC-Exos and hFP-Exos intended to reduce the abundance of pro-inflammatory bacterial, such as Verrucomicrobia, Akkermansia, *A. muciniphila* and *Escherichia coli*, and reestablish the intestinal microbiota homeostasis. The present study demonstrated that hUC-Exos and hFP-Exos have similar therapeutic effects on mice with colitis. and may be used as a new strategy for the treatment of colitis by the application of exosome derived from perinatal tissues. Our results are beneficial for the understanding of the mechanism between T cells and intestinal microbiota in colitis.

## Supplementary Information

**Additional file 1.** **Table S1.** Primers used for real-time quantitative RT-PCR.

**Additional file 2.** **Figure S1.** Uncropped full-length western blots of exosome marker proteins (TSG101and CD63).

## Data Availability

The obtained metagenomic profiles have been uploaded into the NCBI SRA database and are accessible via the Accession Number: PRJNA787378.

## Abbreviations

MSC: Mesenchymal stem cells
IBD: Inflammatory bowel disease
CD: Crohn’s disease
UC: Ulcerative colitis
IL-6: Interleukin-6
TGF-β: Transforming growth factor β
IL-23: Interleukin- 23
DSS: Dextran sodium sulfate
UC: Umbilical cord
FP: Placenta
BCA: Bicinchoninic acid
NTA: Nanoparticle Tracking Video Microscope PMX-120
SDS–PAGE: Sodium dodecyl sulfate–polyacrylamide gel electrophoresis
PVDF: Polyvinylidene difluoride
DSS7D: DSS administered for 7 days and euthanasia
DSS14D: DSS administered for 7 days and kept for another 7 days without any treatment
hUC-Exos: DSS administered for 7 days and followed with hUC-Exos infusion for 7 days
hFP-Exos: DSS administered for 7 days and followed with hFP-Exos infusion for 7 days
PBMC: Peripheral blood mononuclear cell

## References

[1] Ananthakrishnan AN (2015). Epidemiology and risk factors for IBD. Nat Rev Gastroenterol Hepatol.

[2] Eisenstein M (2016). Biology: a slow-motion epidemic. Nature.

[3] Kamm MA (2017). Rapid changes in epidemiology of inflammatory bowel disease. Lancet (London, England).

[4] Zhang YZ, Li YY (2014). Inflammatory bowel disease: pathogenesis. World J Gastroenterol.

[5] Geremia A, Jewell DP (2012). The IL-23/IL-17 pathway in inflammatory bowel disease. Expert Rev Gastroenterol Hepatol.

[6] Zhou L, Ivanov II, Spolski R, Min R, Shenderov K, Egawa T, Levy DE, Leonard WJ, Littman DR (2007). IL-6 programs T(H)-17 cell differentiation by promoting sequential engagement of the IL-21 and IL-23 pathways. Nat Immunol.

[7] Kobayashi T, Okamoto S, Hisamatsu T, Kamada N, Chinen H, Saito R, Kitazume MT, Nakazawa A, Sugita A, Koganei K (2008). IL23 differentially regulates the Th1/Th17 balance in ulcerative colitis and Crohn's disease. Gut.

[8] Sugihara T, Kobori A, Imaeda H, Tsujikawa T, Amagase K, Takeuchi K, Fujiyama Y, Andoh A (2010). The increased mucosal mRNA expressions of complement C3 and interleukin-17 in inflammatory bowel disease. Clin Exp Immunol.

[9] Wing K, Sakaguchi S (2010). Regulatory T cells exert checks and balances on self tolerance and autoimmunity. Nat Immunol.

[10] Carrier Y, Gao W, Korn T, Strom TB, Oukka M, Weiner HL, Kuchroo VK, Bettelli EJN (2006). Reciprocal developmental pathways for the generation of pathogenic effector TH17 and regulatory T cells. Nature.

[11] Yano JM, Yu K, Donaldson GP, Shastri GG, Ann P, Ma L, Nagler CR, Ismagilov RF, Mazmanian SK, Hsiao EY (2015). Indigenous bacteria from the gut microbiota regulate host serotonin biosynthesis. Cell.

[12] Reinhardt C, Bergentall M, Greiner TU, Schaffner F, Ostergren-Lunden G, Petersen LC, Ruf W, Backhed F (2012). Tissue factor and PAR1 promote microbiota-induced intestinal vascular remodelling. Nature.

[13] Scanlan PD, Shanahan F, O'Mahony C, Marchesi JR (2006). Culture-independent analyses of temporal variation of the dominant fecal microbiota and targeted bacterial subgroups in Crohn's disease. J Clin Microbiol.

[14] Ott SJ, Musfeldt M, Wenderoth DF, Hampe J, Brant O, Folsch UR, Timmis KN, Schreiber S (2004). Reduction in diversity of the colonic mucosa associated bacterial microflora in patients with active inflammatory bowel disease. Gut.

[15] Eduard A, Slayden LC, Ching KL, Koch MA, Wolf NK, Plichta DR, Brown EM, Graham DB, Xavier RJ, Moon JJJS (2019). Akkermansia muciniphila induces intestinal adaptive immune responses during homeostasis. Science.

[16] Moll G, Drzeniek N, Kamhieh-Milz J, Geissler S, Volk H-D, Reinke P (2020). MSC therapies for COVID-19: importance of patient coagulopathy, thromboprophylaxis, cell product quality and mode of delivery for treatment safety and efficacy. Front Immunol.

[17] Richardson SM, Kalamegam G, Pushparaj PN, Matta C, Memic A, Khademhosseini A, Mobasheri R, Poletti FL, Hoyland JA, Mobasheri A (2016). Mesenchymal stem cells in regenerative medicine: focus on articular cartilage and intervertebral disc regeneration. Methods.

[18] Roberta B, Raffaella M (2017). ALS pathogenesis and therapeutic approaches: the role of mesenchymal stem cells and extracellular vesicles. Front Cell Neurosci.

[19] Panés J, García-Olmo D, Van Assche G, Frederic J, Lancet CJ (2016). Expanded allogeneic adipose-derived mesenchymal stem cells (Cx601) for complex perianal fistulas in Crohn's disease: a phase 3 randomised, double-blind controlled trial. Lancet.

[20] Mao F, Tu Q, Wang L, Chu FL, Li X, Li HYS, Xu WR (2017). Mesenchymal stem cells and their therapeutic applications in inflammatory bowel disease. Oncotarget.

[21] Kraitchman DL, Tatsumi M, Gilson WD, Ishimori T, Kedziorek D, Walczak P, Segars WP, Chen HH, Fritzges D, Izbudak I (2005). Dynamic imaging of allogeneic mesenchymal stem cells trafficking to myocardial infarction. Circulation.

[22] McBride C, Gaupp D, Phinney DG (2003). Quantifying levels of transplanted murine and human mesenchymal stem cells in vivo by real-time PCR. Cytotherapy.

[23] Wang Y, Chen X, Cao W, Shi Y (2014). Plasticity of mesenchymal stem cells in immunomodulation: pathological and therapeutic implications. Nat Immunol.

[24] Costa LA, Eiro N, Fraile M, Gonzalez LO, Saa J, Garcia-Portabella P, Vega B, Schneider J, Vizoso FJ (2021). Functional heterogeneity of mesenchymal stem cells from natural niches to culture conditions: implications for further clinical uses. Cell Mol Life Sci.

[25] Kamerkar S, LeBleu VS, Sugimoto H, Yang S, Ruivo CF, Melo SA, Lee JJ, Kalluri R (2017). Exosomes facilitate therapeutic targeting of oncogenic KRAS in pancreatic cancer. Nature.

[26] Colombo M, Raposo G, Thery C (2014). Biogenesis, secretion, and intercellular interactions of exosomes and other extracellular vesicles. Annu Rev Cell Dev Biol.

[27] Jacopo B, Silvia M, Gai C, Yonathan G, Sharad K, Giovanni C (2016). Stem cell-derived extracellular vesicles and immune-modulation. Front Cell Dev Biol.

[28] Elahi KC, Gerd K, Meltem AA, Sievert KD, Sheila MN, Aicher WK (2016). Human mesenchymal stromal cells from different sources diverge in their expression of cell surface proteins and display distinct differentiation patterns. Stem Cells Int.

[29] Chen J-Y, Mou X-Z, Du X-C, Xiang C (2015). Comparative analysis of biological characteristics of adult mesenchymal stem cells with different tissue origins. ScienceDirect.

[30] Baglio S, Rooijers K, Koppers-Lalic D, Verweij F, Lanzón M, Zini N, Naaijkens B, Perut F, Niessen HWM, Baldini N, Pegtel DM (2015). Human bone marrow- and adipose-mesenchymal stem cells secrete exosomes enriched in distinctive miRNA and tRNA species. Stem Cell Res Ther.

[31] Okayasu I, Hatakeyama S, Yamada M, Ohkusa T, Inagaki Y, Nakaya R (1990). A novel method in the induction of reliable experimental acute and chronic ulcerative colitis in mice. Gastroenterology.

[32] Jiang B, Fu X, Yan L, Li S, Zhao D, Wang X, Duan Y, Yan Y, Li E, Wu K, Inglis BM, Ji W, Xu R-H, Si W (2008). Transplantation of human ESC-derived mesenchymal stem cell spheroids ameliorates spontaneous osteoarthritis in rhesus macaques. Theranostics.

[33] Cosenza S, Toupet K, Maumus M, Luz-Crawford P, Blanc-Brude O, Jorgensen C, Noël D (2018). Mesenchymal stem cells-derived exosomes are more immunosuppressive than microparticles in inflammatory arthritis. Theranostics.

[34] Maroto R, Zhao Y, Jamaluddin M, Popov VL, Wang H, Kalubowilage M, Zhang Y, Luisi J, Sun H, Christopher T, Stefan H, Motamedi M, Brasier AR (2017). Effects of storage temperature on airway exosome integrity for diagnostic and functional analyses. J Extracell Vesicles.

[35] Cleopatra K, Jeet S, Beyer J, Guerrero S, Lesch J, Wang X, Devoss J, Diehl L (2013). An entirely automated method to score DSS-induced colitis in mice by digital image analysis of pathology slides. Dis Model Mech.

[36] Marjolijn D, Anne C, Helene R, Manon E, Barbara B, Henricus W, Engelina M, Frits K, Jaap J, Herma H, Auke P, Willem E, Gijs R, Daniel W (2010). Autologous bone marrow-derived mesenchymal stromal cell treatment for refractory luminal Crohn's disease: results of a phase I study. Gut.

[37] Dhere T, Copland I, Garcia M, Chiang KY, Chinnadurai R, Prasad M, Galipeau J, Kugathasan S (2016). The safety of autologous and metabolically fit bone marrow mesenchymal stromal cells in medically refractory Crohn's disease: a phase 1 trial with three doses. Aliment Pharmacol Ther.

[38] Tkach M, Théry C (2016). Communication by extracellular vesicles: where we are and where we need to go. Cell.

[39] Wei M, Gao X, Liu L, Li Z, Wan Z, Dong Y, Chen X, Niu Y, Zhang J, Yang G (2020). Visceral adipose tissue derived exosomes exacerbate colitis severity via pro-inflammatory MiRNAs in high fat diet fed mice. ACS Nano.

[40] Chang Y, Zhang Y, Jiang Y, Zhao L, Lv C, Huang Q, Guan J, Jin S (2022). From hair to colon. Hair follicle-derived MSCs alleviate pyroptosis in DSS-induced ulcerative colitis by releasing exosomes in a paracrine manner. Oxidative Med Cell Longev.

[41] Yang R, Liao Y, Wang L, He P, Hu Y, Yuan D, Wu Z, Sun X (2019). Exosomes derived from M2b macrophages attenuate DSS-induced colitis. Front Immunol.

[42] Mao F, Wu Y, Tang X, Kang J, Zhang B, Yan Y, Qian H, Zhang X, Xu W (2017). Exosomes derived from human umbilical cord mesenchymal stem cells relieve inflammatory bowel disease in mice. Biomed Res Int.

[43] Rocchi A, Benchimol EI, Bernstein CN, Bitton A, Feagan B, Panaccione R, Glasgow KW, Fernandes A, Ghosh SJ (2012). Inflammatory bowel disease: a Canadian burden of illness review. Can J Gastroenterol.

[44] Ueno A, Ghosh A, Hung D, Li Ji, Jijon H (2015). Th17 plasticity and its changes associated with inflammatory bowel disease. World J Gastroenterol.

[45] Zielinski CE, Mele F, Aschenbrenner D, Jarrossay D, Sallusto FJN (2012). Pathogen-induced human TH17 cells produce IFN-γ or IL-10 and are regulated by IL-1β. Nature.

[46] McGeachy MJ, Chen Y, Tato CM, Laurence A, Joyce-Shaikh B, Blumenschein WM, McClanahan TK, O'Shea JJ, Cua DJ (2009). The interleukin 23 receptor is essential for the terminal differentiation of interleukin 17-producing effector T helper cells in vivo. Nat Immunol.

[47] Griseri T, McKenzie BS, Schiering C, Powrie F (2012). Dysregulated hematopoietic stem and progenitor cell activity promotes interleukin-23-driven chronic intestinal inflammation. Immunity.

[48] Daniel B, Erik A, Johan H, Carl E, Stephen T, Rahul K, Alex T, Asa V, Mauro D, Fernando G, Jørgen J, Petr R, Jack S, Dirk R, Pontus K, Jonas H, IBD Character Consortium (2021). Systemic inflammation in preclinical ulcerative colitis. Gastroenterology.

[49] Cooney LA, Towery K, Endres J, Fox DA (2011). Sensitivity and resistance to regulation by IL-4 during Th17 maturation. J Immunol.

[50] Blatner NR, Mulcahy MF, Dennis KL, Scholtens D, Khazaie K (2012). Expression of RORγt marks a pathogenic regulatory T cell subset in human colon cancer. Sci Transl Med.

[51] Xavier RJ, Podolsky DK (2007). Unravelling the pathogenesis of inflammatory bowel disease. Nature.

[52] Gareth J, Dominique G, Lynnette R (2013). Why interleukin-10 supplementation does not work in Crohn's disease patients. World J Gastroenterol.

[53] Li B, Gurung P, Malireddi RK, Vogel P, Kanneganti TD, Geiger TL (2015). IL-10 engages macrophages to shift Th17 cytokine dependency and pathogenicity during T-cell-mediated colitis. Nat Commun.

[54] Wilke CM, Wang L, Wei S, Kryczek I, Huang E, Kao J, Lin Y, Fang J, Zou W (2011). Endogenous interleukin-10 constrains Th17 cells in patients with inflammatory bowel disease. J Transl Med.

[55] Sartor RB (2008). Microbial influences in inflammatory bowel diseases. Gastroenterology.

[56] Dicksved J, Halfvarson J, Rosenquist M, Jarnerot G, Tysk C, Apajalahti J, Engstrand L, Jansson JK (2008). Molecular analysis of the gut microbiota of identical twins with Crohn's disease. ISME J.

[57] Kubinak JL, Petersen C, Stephens WZ, Soto R, Bake E, O'Connell RM, Round JL (2015). MyD88 signaling in T cells directs IgA-mediated control of the microbiota to promote health. Cell Host Microbe.

[58] Alexander S, Yvonne D, Vera LB, Christoph G, Önder G, Anne R, Jürgen N, Karsten-Henrich W, Alexander G, Markus B (2017). Reduced mass and diversity of the colonic microbiome in patients with multiple sclerosis and their improvement with ketogenic diet. Front Microbiol.

[59] Kelly A, Madeline B, Tatyana H, Pankaj C, Tommi V, Abhiram S, Jarod P, Archana K, Cary S, Michael E, Glen A, Aleksandar D, Jennifer G, Ramnik J, Subra K (2016). Dysbiosis, inflammation, and response to treatment: a longitudinal study of pediatric subjects with newly diagnosed inflammatory bowel disease. Genome Med.

[60] Conte MP, Schippa S, Zamboni I, Penta M, Chiarini F, Seganti L, Osborn J, Falconieri P, Borrelli O, Cucchiara S (2007). Gut-associated bacterial microbiota in paediatric patients with inflammatory bowel disease. Gut.

