# Supplementary material for: Perinatal tissue-derived exosomes ameliorate colitis in mice by regulating the Foxp3 + Treg cells and gut microbiota
Source: Stem Cell Res Ther. 2023 Mar 20;14:43. doi: 10.1186/s13287-023-03263-1 (PMC10029206; doi:10.1186/s13287-023-03263-1)
Supplement: Supplementary file 1 — Additional file 1. Table S1. Primers used for real-time quantitative RT-PCR. [file 13287_2023_3263_MOESM1_ESM.docx]

Additional file 1: Table S1 The PCR primer sequences

| **Target gene** | **Primer** | **Sequence (5' to 3)** |
| --- | --- | --- |
| IL-6 | M-IL-6-F | TCTATACCACTTCACAAGTCGGA |
|  | M-IL-6-R | GAATTGCCATTGCACAACTCTTT |
| IFN-γ | M-IFN-γ-F | GCCACGGCACAGTCATTGA |
|  | M-IFN-γ-R | TGCTGATGGCCTGATTGTCTT |
| IL-17A | M-IL17-A-F | GGCCCTCAGACTACCTCAAC |
|  | M-IL17-A-R | TCTCGACCCTGAAAGTGAAGG |
| IL-10 | M-IL-10-F | GCTGGACAACATACTGCTAACC |
|  | M-IL-10-R | ATTTCCGATAAGGCTTGGCAA |
| IL-23 | M-IL23-p19-F | AATAATGTGCCCCGTATCCAGT |
|  | M-IL23-p19-R | GCTCCCCTTTGAAGATGTCAG |
| IL-4 | M-IL-4-F | CCCCAGCTAGTTGTCATCCTG |
|  | M-IL-4-R | CAAGTGATTTTTGTCGCATCCG |
