# Supplementary material for: Perinatal tissue-derived exosomes ameliorate colitis in mice by regulating the Foxp3 + Treg cells and gut microbiota
Source: Stem Cell Res Ther. 2023 Mar 20;14:43. doi: 10.1186/s13287-023-03263-1 (PMC10029206; doi:10.1186/s13287-023-03263-1)
Supplement: Supplementary file 2 — Additional file 2. Figure S1. Uncropped full-length western blots of exosome marker proteins (TSG101and CD63). [file 13287_2023_3263_MOESM2_ESM.docx]

Additional file 2

A


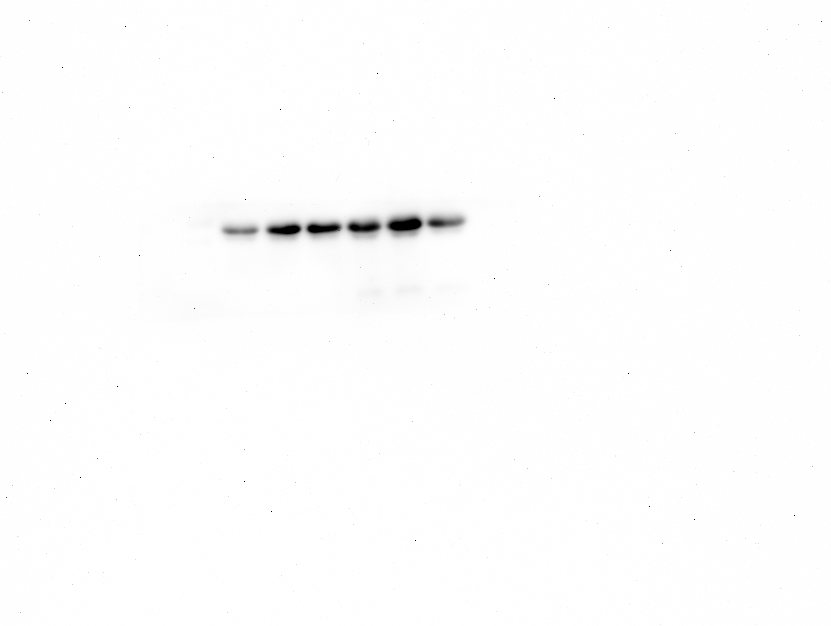


B


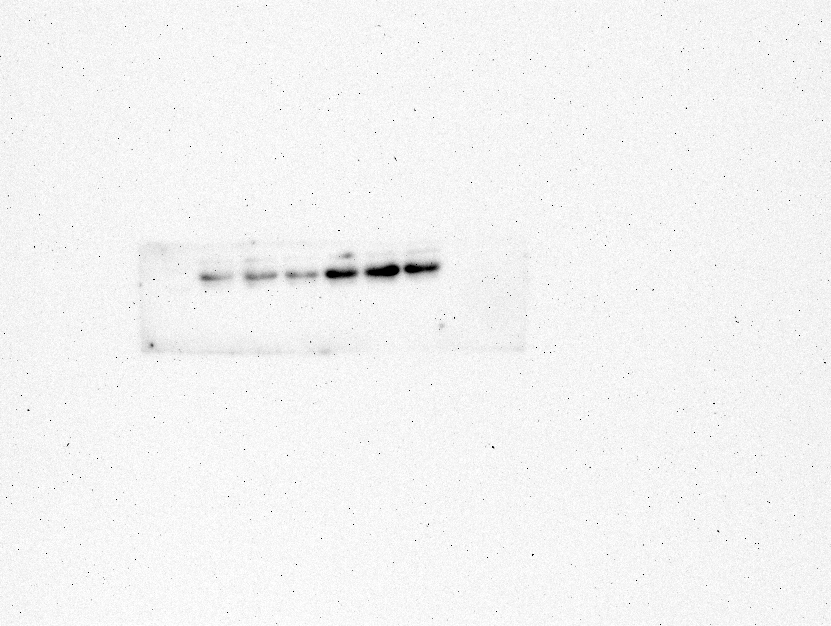


C


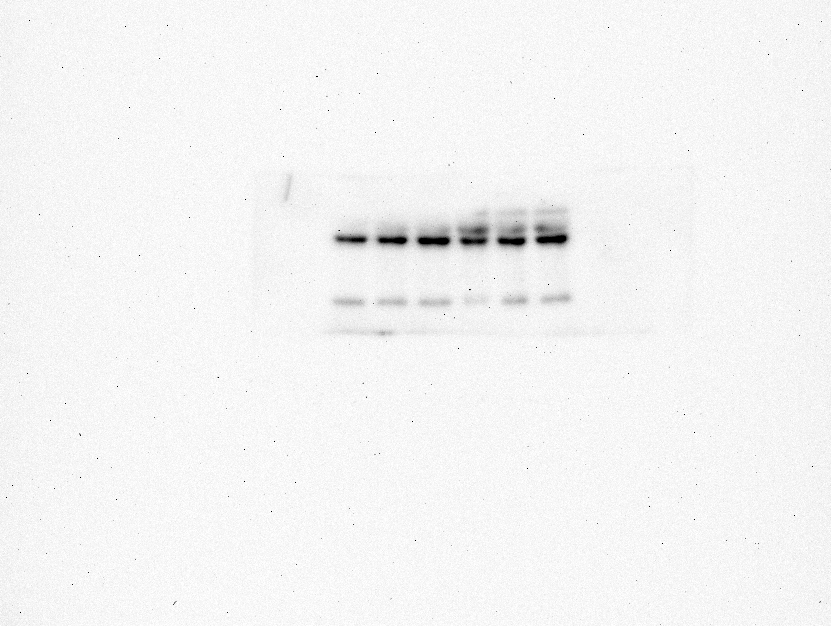


Figure S1 Uncropped full-length western blots. (A) TSG101. (B) CD63. (C) Beta-actin. The red box represents the clipping position in the main text.
